# Supplementary figures and images for: Modulation transfer functions for audiovisual speech
Source: PLoS Comput Biol. 2022 Jul 19;18(7):e1010273. doi: 10.1371/journal.pcbi.1010273 (PMC9295967; doi:10.1371/journal.pcbi.1010273)

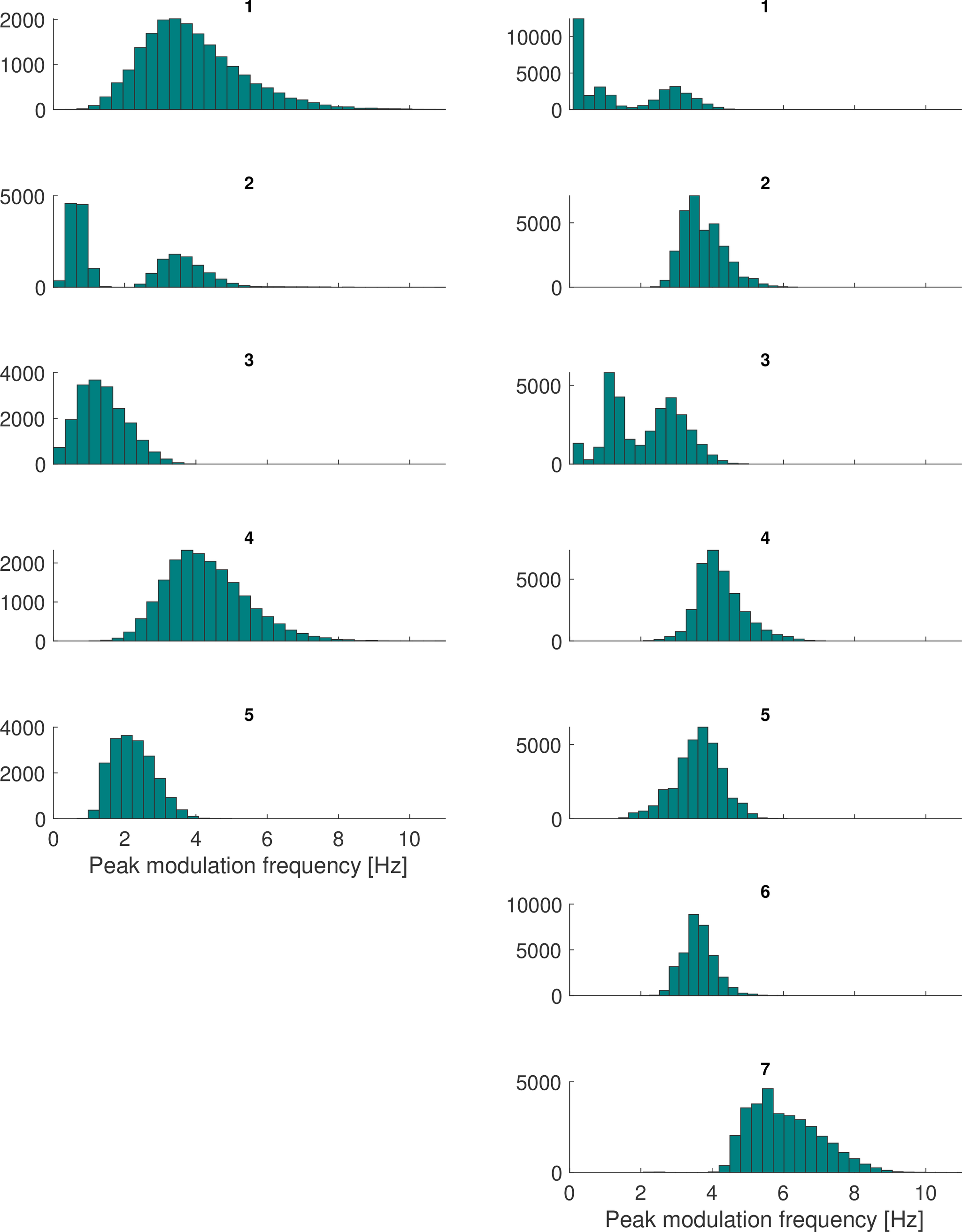

Supplement: S1 Fig — The variation in spectral peaks across videos aligns with the shape of the CCA-derived modulation filters (Figs 2 and 4). (TIF) [file pcbi.1010273.s002.tif]

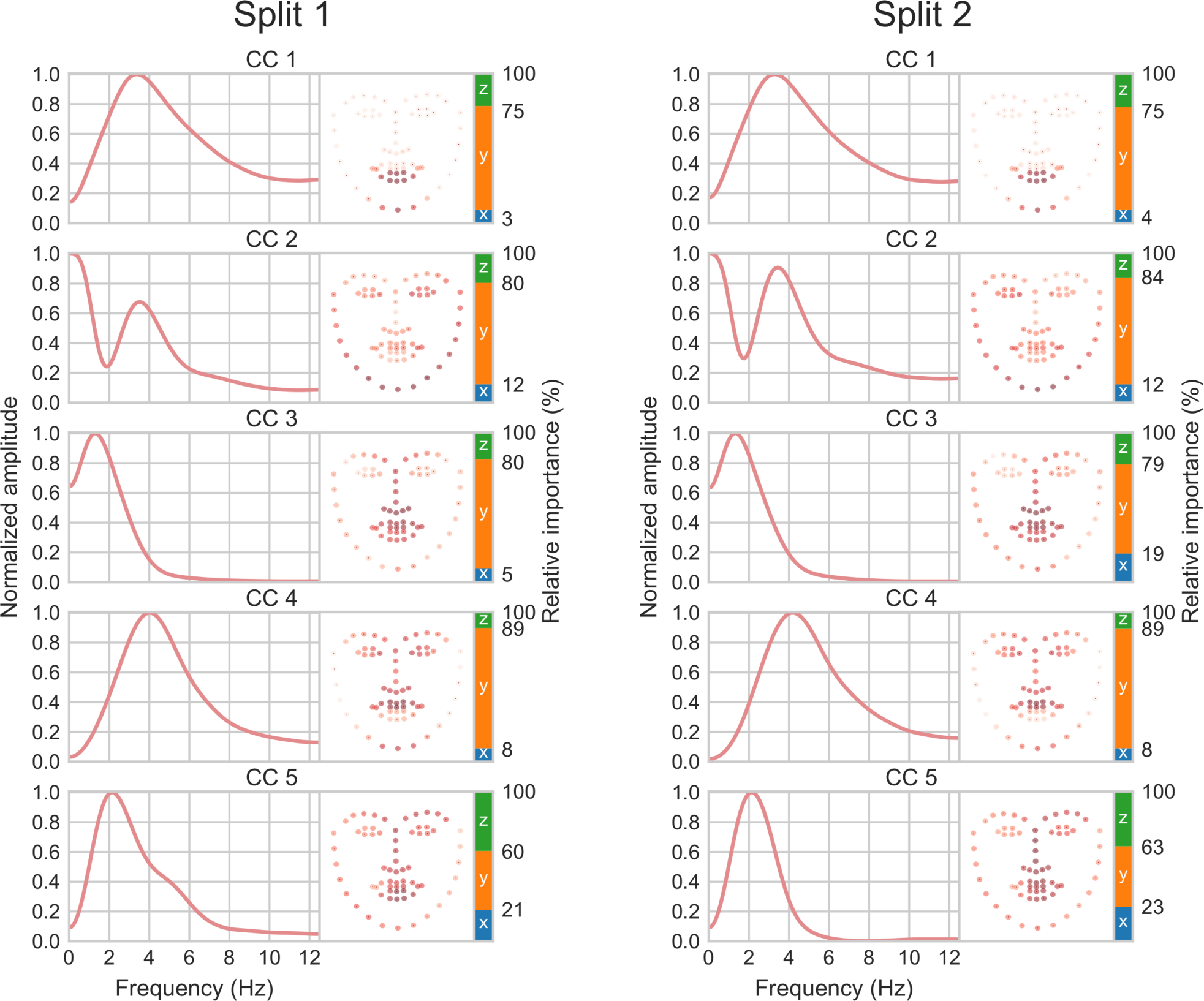

Supplement: S2 Fig — The same CCA analysis was performed on two independent halves of the LRS3 dataset (∼1950 different speakers in each split). Envelope filters (left panels) and spatial decompositions of the visual face (right panels) learned via CCA were highly similar between the two data splits. (TIF) [file pcbi.1010273.s003.tif]

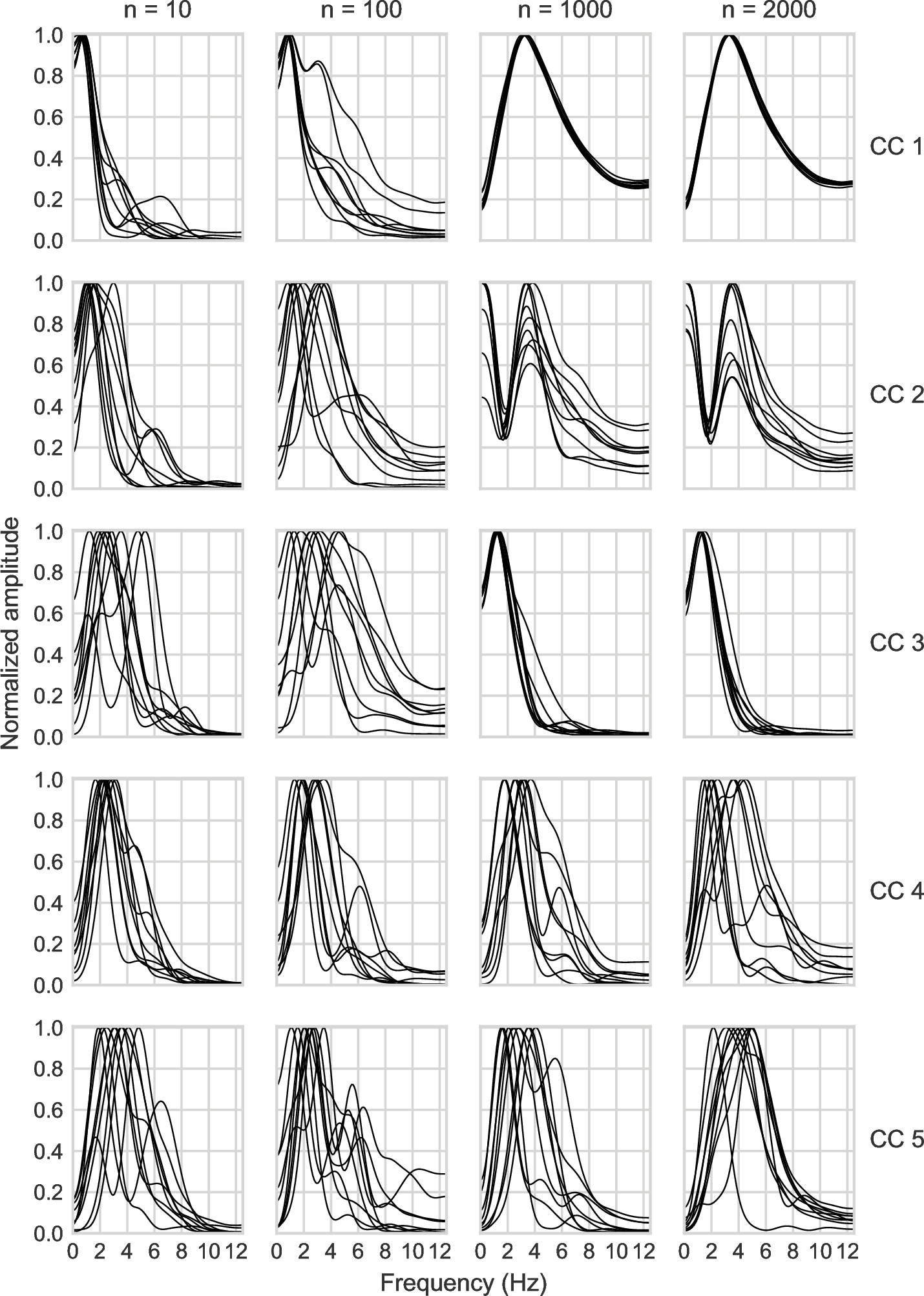

Supplement: S3 Fig — MTFs were computed for different amounts of speakers by subsampling the data. For different numbers of speakers, nine different CCA solutions were computed while keeping regularisation parameters fixed. As can be seen, a higher number of speakers lead to more convergent solutions. We note that CCs 4 and 5 may switch place in different subsamples. (TIF) [file pcbi.1010273.s004.tif]

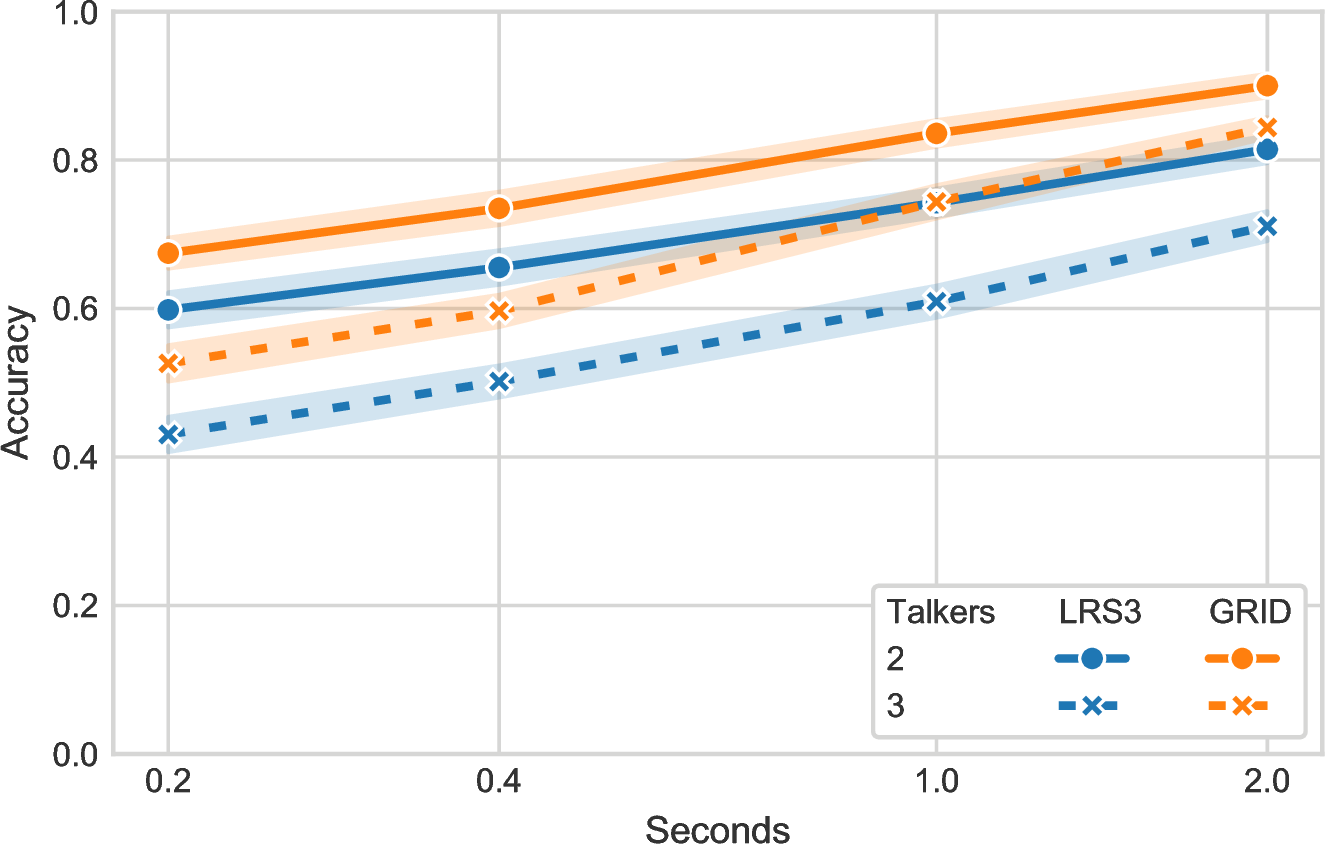

Supplement: S4 Fig — The AV CCA model enables fast speaker identification. Here, the CCA model is used to identify which of 2 (solid lines) or 3 (dashed lines) different audio segments correspond to 2 or 3 video segments. The AV pair with the highest correlation on CC1 is chosen as the matching pair. Only videos not used for training the CCA model were used for speaker identification. Identification performance is shown as a function of AV segment duration for the LRS3 (blue) and GRID (orange) data. Shaded regions show ± SEM. (TIF) [file pcbi.1010273.s005.tif]

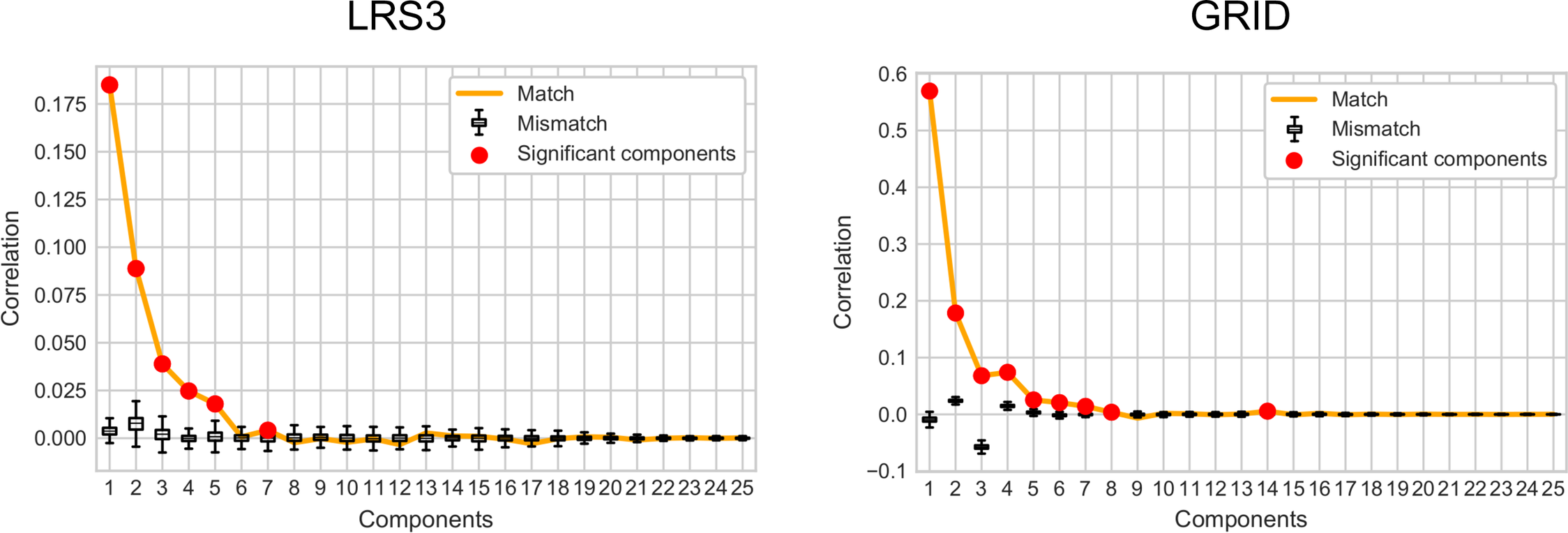

Supplement: S5 Fig — Test correlation values for the LRS3 (left) and GRID data (right). Boxes show null distributions derived by training CCA models on mismatching AV data. (TIF) [file pcbi.1010273.s006.tif]
