# Supplementary material for: Modulation transfer functions for audiovisual speech
Source: PLoS Comput Biol. 2022 Jul 19;18(7):e1010273. doi: 10.1371/journal.pcbi.1010273 (PMC9295967; doi:10.1371/journal.pcbi.1010273)
Supplement: Attachment — Submitted filename: Response.docx [file pcbi.1010273.s007.docx]

We would like to thank both reviewers for their positive feedback and constructive comments. They have helped us improve the paper. We highly appreciate their time and effort revising our work. Below we have inserted our responses to the individual comments in blue.

Reviewer #1: This impressive manuscript presents results from the use of regularized canonical correlation analysis on speech acoustics and video to learn temporal envelope filters that correlate with motion on different parts of the face. In both machine and human recognition of speech integration of audio and visual information from talkers enhances performance. This research provides an innovative and rigorous methodology to address part of this problem: What is the linear correspondence between the two signals?

The strengths of the work include that it analyzes a large AV data set as well as a more controlled smaller data set, a broad literature review considering the many issues (though a few key references noted below should be considered), and rigorous methods that consider the joint reduction in dimensionality for auditory and video signals to compute the correlated regions and timescales.

References to consider:
On the relationship between face movements, tongue movements, and speech acoustics
J Jiang, A Alwan, PA Keating, ET Auer, LE Bernstein - EURASIP Journal on Advances in Signal Processing, 2002

Quantifying time-varying coordination of multimodal speech signals using correlation map analysis
A Vilela Barbosa, RM Déchaine, E Vatikiotis-Bateson… - The Journal of the Acoustical Society of America, 2012

Grant, K. W., and Seitz, P. F. (2000). “The use of visible speech cues for improving auditory detection of spoken sentences,” J. Acoust. Soc. Am. 108(3), 1197–1208.
Yehia, H., Rubin, P., and Vatikiotis-Bateson, E. (1998). ‘‘Quantitative association
of vocal-tract and facial behavior,’’ Speech Commun. 26, 23–43.

Thank you for your comments and these valuable references. Yehia et al. (1998) was a critical miss. We have added these references to the revised MS.

The weaknesses of the manuscript include: A) In spite of the large data set and improved methods, the results are not really surprising. As the omitted references suggest, there are visual correlations between the acoustic envelope and parts of the moving head and face. The partition shown in this paper is nice but is partially anticipated by other studies that show additional correlation features. However, demonstrating something that is not surprising is still worth doing.

We agree, the results themselves are consistent with previous work. We also believe that the large-scale data analysis and the CCA filtering approach is still worth doing. The previous studies are typically based on motion data from few subjects (<15, often only 2) and studies usually fit the AV data per talker. We address the critical question of generalizability across talkers and the robustness of AV speech statistics across talkers. Indeed, we see that across-talker filters become stable with larger amounts of talkers (S3 Fig). Although previous studies also relate envelopes and the moving head, we are not aware of work that quantifies the transfer function of the envelope, i.e. a method that decomposes the envelope to find the modulation rates that are correlated with the visual component. This method itself may also be useful for others and of general interest.

B) The authors discuss a number of limitations but one that is inherent to all approaches like this is that components are dominated by larger variances sources. However, information that is perceptually salient can be quite small and statistically infrequent, but that information can make all the difference. For example, good lipreaders take advantage of high spatial frequency visual information in some contexts even though low spatial frequency aspects of the signal are generally fine.

We agree. Our analysis focuses specifically on AV signal statistics that can be extracted from large-scale video datasets and are predictive across speakers. The relevance to perception is not given and it is clear that many cues that are perceptually important in AV speech are not captured in this analysis. The comment also relates to an important general point about the inherent bias towards large-variance sources in our approach. In our analysis method, we explicitly focus only on AV statistics that generalize between the many speakers in the datasets. There is an inherent trade-off between generalizability and the amount of variance in the AV data that can be explained. Models fit on individual speakers typically yield much higher correlation, especially if the correlation is reported on the same data, but may fail to generalize to other speakers. Forcing the CCA to generalize across many speakers with natural speech implies that mainly relative ‘coarse’ features with high variance will be revealed. We fully acknowledge that many more subtle features are highly important in AV speech perception. We have inserted an additional paragraph in the ‘limitations’ section to further stress this important point.

C) It seems that the correlations are computed on segments, thus with stationary AV temporal alignment. As the authors suggest temporal synchrony is a complex problem but perhaps more complex than they suggest. There are differences in physical transmission of speech sight and sound as well as differences in neural transmission and processing in the two modalities. But, also the two speech signals differ greatly in character. Acoustic speech can be segmented with more or less accuracy because it is punctuated by syllabic nuclei and silences. Visual speech is far more continuous and segmenting it is more similar to the problem of visual event perception (e.g., Zacks, 2020). In the past, this problem has been hacked by omitting the signals associated with silences (e.g., Yehia). This just avoids the problem.

We acknowledge this limitation. Our analysis is limited to stationary coupling of temporal envelope features. We have investigated potential time-varying correlations with temporal kernel CCA (similar to Bießmann et al. 2010 or the CCA analysis in de Cheveigné at al 2018). In short, we augment either the visual or audio feature with time-lagged features and perform the regularized CCA. This results in a temporal FIR filter reflecting potential time-shifted (linear) couplings between the audio and video features. This analysis did not improve our results and did not reveal any robust time-lag correlations. We interpret this as indicating that the relatively coarse temporal envelope features that are predictive across speakers are not sensitive to such time shifts. Low-pass filtered temporal envelopes may largely absorb time-shifts. Other acoustic features that are important in AV perception may well display complex temporal dependencies, but may be difficult to capture in such large-scale across-speaker analysis of natural speech. This could nonetheless be pursued further, and we are making all the data available for future studies.

Bießmann, Felix, et al. "Temporal kernel CCA and its application in multimodal neuronal data analysis." *Machine Learning* 79.1 (2010): 5-27.

de Cheveigné, Alain, et al. "Decoding the auditory brain with canonical component analysis." *NeuroImage* 172 (2018): 206-216.

D) The temporal signal compression to match video and acoustic signals predetermines that only low frequency envelope filters will be observed. Granted that there can’t be much spectral power in the movements that is perceptually important (see de Paula et al.).

H. de Paula, H.C. Yehia, D. Shiller, G. Jozan, K.G. Munhall, E.Vatikiotis-Bateson
Linking production and perception through spatial and temporal filtering of visible speech information
Proceedings of the 6th International Seminar on Speech Production, Sydney, Australia, 7–10 December 2003 (2003), pp. 37-42.

But there is information in the acoustics above this low frequency filter level that can be predictive of movement and vice versa (e.g., in a Bayesian manner). In other words, correlation of temporally matched signals is not the only kind of binding.

Agreed. Paula et al. and also, e.g., Munhall et al. 1998 suggest that information below 10 Hz is salient and important for speech intelligibility. Our focus here was explicitly on the temporal envelope at low frequencies, but that does not imply that high-frequency information and/or complex temporal couplings of cues are not important. Other feature representations apart from envelope-based ones could be pursued. We have also explored MFCCs, that tend to yield higher prediction accuracies. Again, we only demonstrate that low-frequency temporal envelope statistics is an important generalizable feature of AV speech signals, not that it is exclusive. Our method is based on a decomposition in the envelope domain to derive MTFs. Other cues would be highly relevant to investigate but we feel that it would shift the focus of the current paper. We also comment on these points in the revised manuscript (‘limitations’ section).

Some additional comments:

Line 250… This is a possible null distribution but of all of the control distributions that could be computed this is certainly the weakest. Given the differences in speaking style and rate between talkers (as the authors show), this will produce a very uncorrelated distribution. But, if you wanted to control for differences in speaking rate and emphatic variance in speech, you could use utterances from the same talker or the same utterance played in reverse. I am not suggesting that this be done but be aware that this is a very null null. Perhaps the null distribution and the data correlations could be shown graphically in an appendix. This would make me understand the sentence on line 254-255. By the way, how many matching components were discarded as a result of that criterion.

We have added a plot of the data correlations and null distributions to the appendix (S5 Fig) as suggested. S5 Fig shows the null distributions derived from CCA models trained on mismatching data. The fact that the null is based on mismatching segments from other talkers is, in fact, not so critical. As discussed above, our across-speaker approach identifies robust but rather coarse features that will be similarly (un)correlated for temporally mismatching segments of the same talker. To illustrate this, here below we plot correlation null distributions for mismatching segments from the same speaker and from different speakers. Here, a CCA was computed on matching segments on the first 1000 videos of the lrs3 dataset. We then compute test correlations for CC1 on held-out test videos for matching (blue) and mismatching (orange) segments, where mismatching segments are either from the same speaker (above) or from different speakers (below). As can be seen, the two mismatch distributions are very similar.

Line 277 – correlations above 1%... What does that mean? And what percentage of the variance does correlation account for overall?

We report only CCs that are statistically significant meaning that they exceed the 95^th^ percentile of the empirical null. We set a lower threshold and do not report CCs where the correlation is below ρ=0.01 (i.e. 1%). We have added a comment to clarify this. Since the data are normalized the correlation values scale with the percentage of variance explained.

S3 Fig. Thus, the shape and peak of the group filters are determined by averaging smear. Note that the individual filters shown here are also averages for individuals’ speech sample and people change rate dynamically even within a phrase.

The large amount of data has a regularizing effect, but it is not a simple averaging smear. Using a smaller amount of data (the leftmost columns in S3 Fig) shows that solutions vary in terms of their spectral filter characteristics, most likely because the solutions pick up differences in speech rate etc. Solutions for larger amounts of data does not smear across this variation. In fact, solutions become more robust and filter outputs more narrowly peaked with more data. We interpret that this as implying that the data amount constraints solutions to those that truly generalize across speakers. Still, the shape of the filters does appear to reflect variation in e.g. speaking rate as we comment on.

Line 359-361. I am happy to see this demonstration about the GRID type tasks being different from more natural speech. I presume that the lack of envelope rates in the 1-2 Hz range for the GRID tasks has something to do with them having no variation in prosody. The TED talks are more natural but are heavily scripted with edited retakes.

We agree with this. It is true that although TED talks have much more natural variation in prosody, they still only represent a subset of the variation that can be encountered in natural speech. We have included a comment acknowledging this fact in the revised MS.

Lines 392-394. I am surprised that the envelope modulations in the two ranges were mutually uncorrelated. They should be related as stress and emphasis signalled by head motions should correlated with mouth aperture size. The different event rate and information span must wash that out.

We were simply referring to the fact that CCA seeks uncorrelated components. CCs computed for individual samples will indeed rarely have zero correlation. We have removed this sentence to avoid confusion.

Lines 376-379. This supports my view that analyses such as this are biased towards uncovering large movement variance as the comment on heart rate and breathing indicates.

Indeed. We also commented on this large-variance bias in the comments above and in the revision.

In summary, this is a well carried out and thoughtful piece of work. It has sophisticated methodology and a number of good controls have been carried out to test alternative explanations. I feel that this manuscript will of interest to researchers in speech recognition and those who study the behavioral and neural concomitants of multisensory speech.

Thank you very much for your time and helpful comments on our paper.

Reviewer #2: Review of Modulation transfer functions for audiovisual speech

In this excellent study, Pedersen et al. revisit the question of the link between auditory and visual cues in Speech. This is a well-studied and important field that has had extensive amount of work. The known result is that visual and auditory cues are roughly correlated in 3-8 Hz frequency bands and that head movement is often in the 1-2 Hz region and is important for speech perception. However, what is missing is a precise quantification of the precise rates at which envelope information is synchronized with motion in different parts of the face.

To address this question, the authors combined a novel approach that first used a deep learning approach to identify face landmarks, and a gammatone filter bank to filter the auditory signals. They then used regularized canonical correlation analysis (rCCA) to learn speech envelope filters whose outputs correlate with motion in different parts of the speakers face. In general, I found the paper to be well written and has some interesting conclusions, albeit consistent with prior work. I had a few comments which will hopefully improve the manuscript.

Statistical significance of components: Could the authors add what correlation is explained by each of the CCs? The authors say they report CCs where correlation was above 1%. That seems quite low.

Thank you. We have added a figure (S5 Fig) showing the correlation values. The statistical significance of each component is determined by the empirical null distribution, derived from training CCA models on mismatching AV data. 1% is not a significance threshold, we simply ignore CCs if they have correlation below 1% even if they are statistically significant. We have clarified this in the revised paper.

Indeed, the correlation values are relatively low compared to analyses based on few talkers and more controlled recordings, as we comment on in the paper and in response to reviewer 1 above. Our approach focuses on finding linear AV correlation components that generalize across speakers. The correlation values are the predicted AV correlations on a separate test set not used for fitting the CCA. Fitting CCA on few individual videos and assessing correlations on the same data yields much higher correlation values. For instance, if we fit CCA models per video with our features, then the CC1 correlation on the same videos will always be close to 1 (perfect correlation), simply because the feature space is relatively high-dimensional (i.e. overfitting). Instead, we fit CCA models across many speakers, and assess the correlation on many other speakers. This across-speaker approach has a strong regularizing effect on the CCA solutions. This results in lower correlation values but finds more robust (more generalizable) AV speech statistics.

We have also, in a follow up analysis, used deep neural networks to align the audio and video data, which results in much higher AV correlations (while still being generalizable across speakers) but at the important expense that the (nonlinear) model weights are no longer directly interpretable. Linear CCA in the current study allows us to derive filters directly in the envelope domain, which we believe is a substantial benefit for interpretation of the results.

Second, I feel the authors are not doing justice to their incredible 4000 speaker dataset by only showing the average correlation. I feel S1 Fig should perhaps be a side panel to the figure 1 and figure 1 should have errorbars around it. Reporting the correlation coefficient next to the canonical components would help the reader. The normalization also makes it hard to assess if some components are larger or smaller. I would say the authors should also include statistics and errorbars for the GRID corpus dataset.

We have revised S1 Fig to include this information, now also showing variation for the GRID data. We also include the new figure (S5 Fig) showing the correlations, also giving an idea of the size of the individual CCs. Since we are analyzing a very high number of videos, errorbars of standard errors on the component plots (e.g. Figs 2 or 4) are so small that they are not visible! However, the subsampling of the data shown in S3 Fig. visualizes the variation in the components when the data are split into different subsets.

Thank you for your helpful comments.
